# An Extracranial, Infratemporal Meningioma Following Neoadjuvant Radiotherapy: A Case Report

**DOI:** 10.7759/cureus.86612

**Published:** 2025-06-23

**Authors:** Luke Borg, David Borg, Bernard Galea, Herman Borg Xuereb

**Affiliations:** 1 Surgery, Mater Dei Hospital, Msida, MLT; 2 Otolaryngology - Head and Neck Surgery, Mater Dei Hospital, Msida, MLT

**Keywords:** extracranial meningioma, infra temporal fossa, infratemporal fossa approach, meningioma, radiotherapy (rt)

## Abstract

Extracranial meningiomas are uncommon tumors and represent an unusual manifestation of a typically intracranial disease. While most are intracranial, primary extracranial presentations may arise independently or represent extensions of intracranial disease. Radiation exposure is a recognized but uncommon risk factor. We present a case of a 66-year-old male patient who developed a painless, preauricular mass more than a decade after cranial radiotherapy for acromegaly. Imaging revealed a well-circumscribed, enhancing lesion centered in the infratemporal fossa, with bony remodeling but no intracranial extension. Surgical excision was performed using a modified Blair approach, preserving the facial nerve. Histology confirmed a WHO Grade I meningioma. This case highlights the importance of long-term follow-up in irradiated patients and the value of multidisciplinary planning for complex skull base tumors.

## Introduction

Meningiomas are typically benign tumors of the central nervous system arising from arachnoid cap cells [1]. Although most occur intracranially, approximately 1-2% may arise extracranially, either as extensions of intracranial tumors or as primary lesions [2,3]. These extracranial meningiomas are thought to develop from ectopic arachnoid cells or undifferentiated mesenchymal tissues [4,5].

Radiation-induced meningiomas (RIMs) are an uncommon but documented long-term complication of cranial irradiation, more commonly affecting children due to increased radiosensitivity, but also seen in adults [6]. The risk increases with cumulative doses exceeding 10-20 Gy, and these tumors may present after latency periods of 10-30 years [7]. A systematic review by Yamanaka et al. found a mean latency of 22.9 years and an average dose of 38.8 Gy among 251 RIM cases [8]. Although frequently aggressive and intracranial, benign extracranial forms remain exceptionally rare [4,5].

We describe a rare case of an extracranial infratemporal meningioma diagnosed more than a decade after cranial radiotherapy for acromegaly. This case underscores the need for long-term imaging surveillance and highlights key diagnostic and surgical considerations in previously irradiated patients.

## Case presentation

A 66-year-old male patient presented with a two-year history of a painless, slowly enlarging mass in the left preauricular region. His medical history included transsphenoidal hypophysectomy for acromegaly, followed by adjuvant cranial radiotherapy with a total dose of 45 Gy administered 12 years earlier. On examination, the mass was firm, non-tender, and located above the zygomatic arch, with no associated neurological or otological symptoms.

Contrast-enhanced MRI revealed a 3.8 × 3.5 × 6.35 cm avidly enhancing soft tissue mass centered on the left temporalis muscle within the masticator space. The lesion abutted the greater wing of the sphenoid with associated bony remodeling, without intracranial extension. A CT confirmed periosteal bone changes but no destruction.

**Figure 1:**
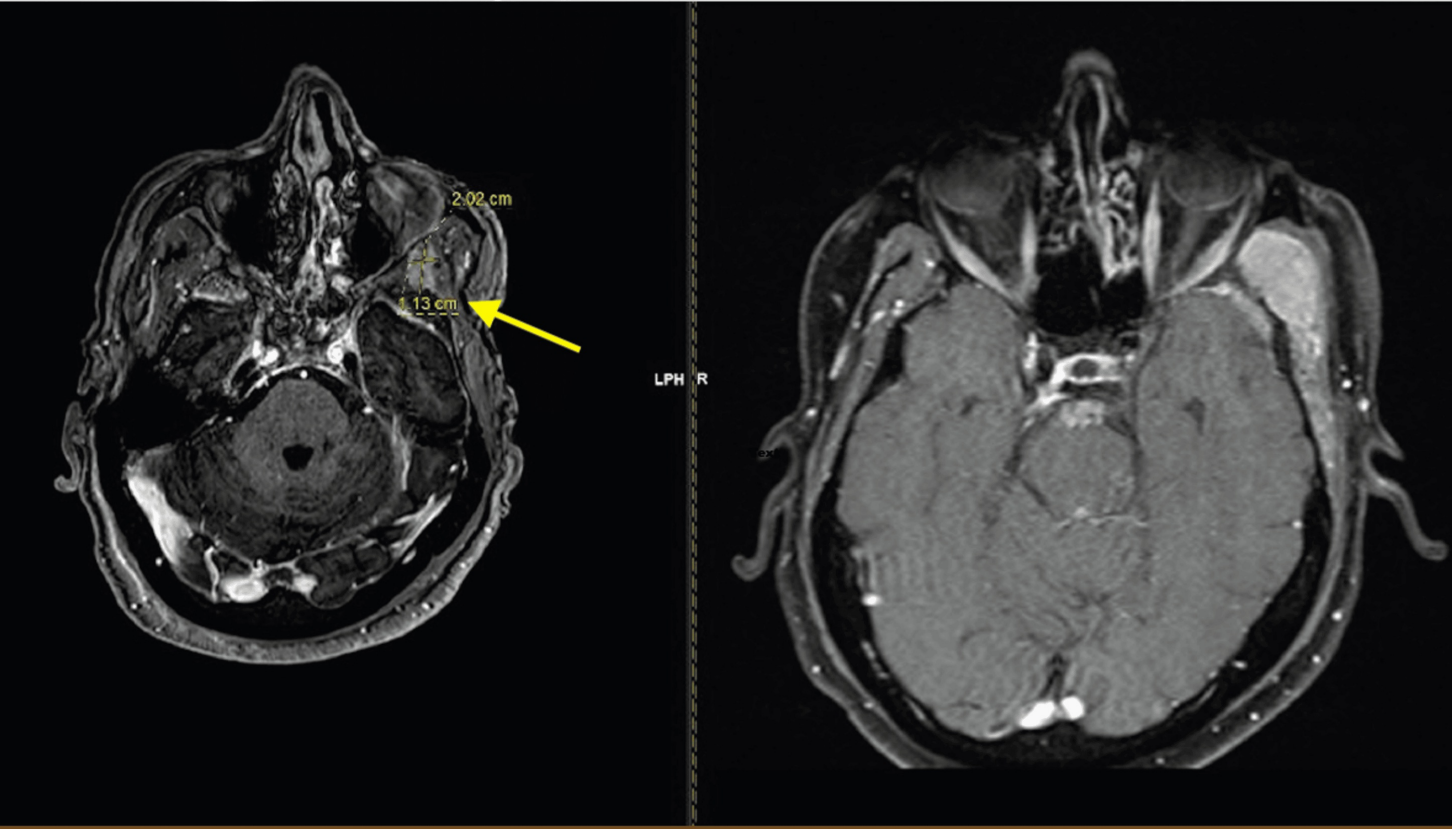
Axial MRI imaging at eight months, growth increased to 5 × 4 × 7.2 cm On the left (labelled in yellow and indicated by the yellow arrow) is the documented lesion described in the manuscript. With fat suppression (on the right), this can be better delineated and differentiated from surrounding tissue.

Eight months later, an MRI showed that the lesion had grown to 5 × 4 × 7.2 cm (Figure 1).

The patient remained asymptomatic, and the multidisciplinary team (MDT) consensus recommended surgical excision. 

Surgical management

A modified Blair incision with a temporal extension provided access. The tumor was centered on the squamous portion of the temporal bone, enveloped by the temporalis and pterygoid muscles. Facial nerve monitoring was used intraoperatively, and the tumor was excised completely with its capsule. The posterior branch of the greater auricular nerve was sacrificed for exposure.

**Figure 2:**
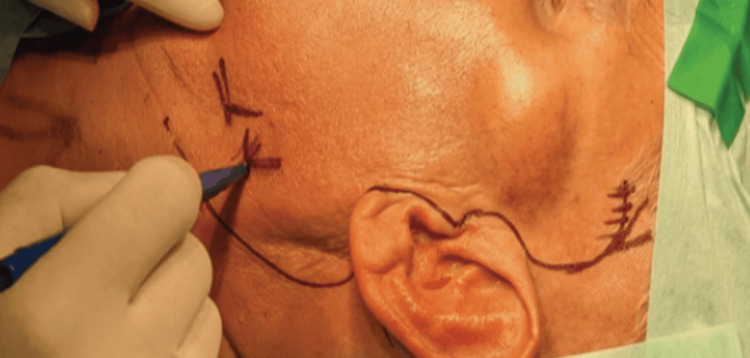
Surgical planning Pre-operative planning included a pre-auricular modified Blair incision with an inferior extension.

Following meticulous excision, the tumor was completely removed with an intact capsule and surrounding medial and lateral pterygoid muscles. The facial nerve was positively identified and stimulated with facial nerve monitor. It was preserved. Figure 2 shows the initial planned incision.

**Figure 3:**
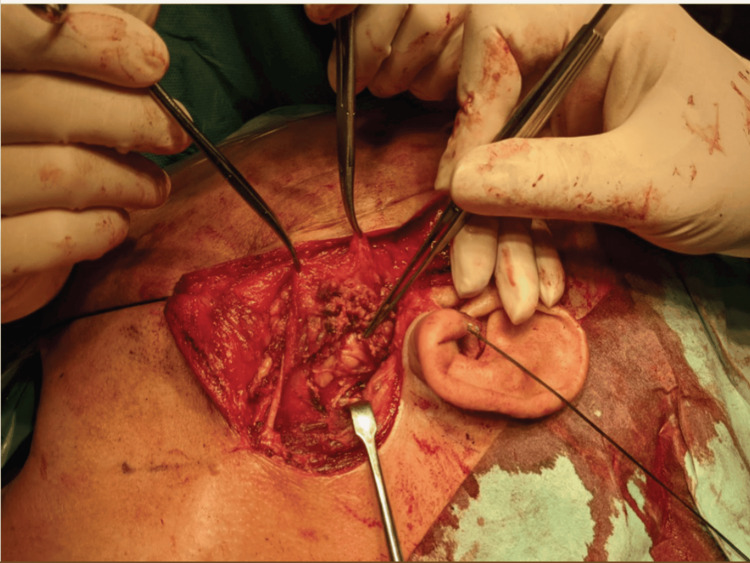
Infratemporal fossal approach Here one can appreciate the meningioma held by the fine non-toothed forceps and its vicinity to the lower branches of the facial nerve as seen after elevation of pre-auricular skin flap.

Figure 3 shows the infratemporal fossa approach after elevation of the skin flap exposing the lower branches of the facial nerve and the adjacent tumor.

Histopathology

Histological analysis revealed a WHO Grade I meningioma composed of spindle cells and psammoma bodies, with no atypia or mitoses [2].

Postoperative course

The patient had a good recovery with preserved facial nerve function (House-Brackmann I [9]) and paraesthesia in the auricular nerve territory. An MRI at one year demonstrated a 1.9 × 3.1 × 4.5 cm postoperative mass at the surgical site. An MDT assessment favored a postoperative change over recurrence, and surveillance was continued.

## Discussion

Extracranial meningiomas are rare and may mimic other soft tissue tumors. These lesions constitute less than 2% of all meningiomas and are defined as those that occur outside the cranial vault without any radiological or intraoperative evidence of continuity with intracranial dura [4]. They are believed to arise from ectopic arachnoid cells, perineural cells, or pluripotent mesenchymal progenitors misplaced during embryological development [4,5,10]. This pathogenesis explains their occurrence in regions distant from the dura, including extracranial soft tissues.

The most common extracranial locations include the calvarium (25%), the infratemporal fossa (22%), the orbit and sinonasal region (15%), and the neck or parapharyngeal space (10%) [4,5]. Less frequent sites include the lungs, shoulder girdle, and retroperitoneum [7,8,10]. Due to their indolent growth and nonspecific presentation, such as a painless mass, diagnosis can be significantly delayed, especially in the absence of neurologic or systemic symptoms [5].

Radiotherapy is a recognized etiological factor for the development of meningiomas. RIMs typically occur after a latency period of 10 to 30 years and are more common in patients who received cranial irradiation during childhood or adolescence due to the increased radiosensitivity of immature neural tissues [11,12]. Adults, however, are not immune to this risk. The incidence of RIM has been reported in approximately 0.1%-2.7% of patients who have received cranial radiotherapy, with some reports estimating an 18-fold increased risk compared to the general population [13,14].

The cumulative radiation dose plays a critical role in determining risk. Doses above 10-20 Gy are associated with increased incidence of RIM, although tumors have been documented following both low-dose (<10 Gy) and high-dose (>40 Gy) exposures [11,12,15]. In a large meta-analysis of 251 patients with RIM, the average latency period was 22.9 years, and the mean radiation dose was 38.8 Gy [11]. While many RIMs are histologically atypical or anaplastic, benign WHO Grade I tumors have also been described, as in our case [2,11,13].

Infratemporal fossa involvement in extracranial meningiomas is particularly challenging due to its proximity to cranial nerves and major vascular structures. The region accounts for approximately 22% of reported extracranial meningioma cases and often necessitates skull base surgical expertise [4,5]. Surgical excision remains the mainstay of treatment, with complete resection offering excellent control rates. Facial nerve preservation is a major concern, particularly in lesions extending to the lateral skull base. The use of intraoperative nerve monitoring, as employed in our case, minimizes iatrogenic nerve injury and improves postoperative outcomes [5,16].

This case underscores several essential points: the importance of long-term follow-up in irradiated patients; the possibility of benign, slow-growing tumors presenting years after treatment; and the need for multidisciplinary planning in managing rare skull base neoplasms. As more patients survive longer after radiotherapy for pituitary or other cranial tumors, clinicians should remain vigilant for delayed neoplastic complications [11,15,17].

## Conclusions

This case highlights a rare presentation of a benign extracranial meningioma in the infratemporal fossa, occurring more than a decade after cranial radiotherapy. Such presentations underscore the importance of maintaining long-term imaging surveillance in patients with prior cranial irradiation, even in the absence of neurological symptoms.

Multidisciplinary decision-making, careful surgical planning, and the use of intraoperative nerve monitoring are critical in managing tumors in anatomically complex regions like the infratemporal fossa. Preoperative counseling is essential to inform patients about potential risks, including injury to neural structures and the possibility of recurrence, which may necessitate further surgical intervention. Such patient education is paramount in delivering high-quality, patient-centered care. 
